# Thank You to Reviewers 2026

**DOI:** 10.3322/caac.70108

**Published:** 2026-08-28

In order to maintain the high standards of *CA*’s content, the Editors of *CA* rely on the knowledge and dedication of many experts in deciding which topics to pursue, which manuscripts to publish, and what modifications to make to ensure medical and scientific accuracy and suitability for our readers. We thank our Associate Editors and our Editorial Advisory Board, who continue to provide these services for us time and time again.

We are also greatly indebted to the effort and expertise of the following individuals for reviewing manuscripts for the journal from July 1, 2025, to June 30, 2026. These individuals go beyond expectations by consistently and expeditiously delivering comprehensive, discerning reviews.

Nadeem R. Abu‐Rustum

Pedro Barata

Surinder Batra

Beth Beadle

Daniel Benedetti

Sushil Beriwal

Stephanie Blank

Susana Campos

Fumiko Chino

Clay Cockerell

Adil Daud

Michael Davies

Ajay Dhakal

Jessica DiSilvestro

Milana Dolezal

Julien Edeline

Bita Esmaeli

Douglas Evans

Vaia Florou

Zhi Ven Fong

Anand Ganesan

Ben George

Amandeep Godara

Krishna Gunturu

Lindsay Hannan

Jeremy Harris

Kate Hetherington

Kathleen Horst

Fernando Hugo

Ahmedin Jemal

Rachel Jimenez

Corinne Joshu

Hina Khan

Albert E. Kim

Kevin B. Kim

Matthew Kudek

Matteo Lambertini

Phebe Lemert

Gilberto Lopes de Lima Lopes Junior

Stefano Luminari

Martin Ma

Kim Margolin

Carol Marquez

Jonathan Marron

Claudia Massarotti

Paul Mathew

Clarissa Mathias

Sandy Mazzoni

Krishnan Patel

Mack Roach

Ilana Schlam

Vivek Subbiah

Molly Taylor

Derek Tsang

Angela E. Usher

Hao Wang

Lori Wirth

Fabio Ynoe de Moraes

Youssef Zeidan

Mu‐Sheng Zeng

Janie Zhang

